# Intestinal GPR119 activation by microbiota-derived metabolites impacts feeding behavior and energy metabolism

**DOI:** 10.1016/j.molmet.2022.101649

**Published:** 2022-11-30

**Authors:** Miki Igarashi, Tetsuhiko Hayakawa, Haruka Tanabe, Keita Watanabe, Akari Nishida, Ikuo Kimura

**Affiliations:** 1 Department of Applied Biological Science, Graduate School of Agriculture, Tokyo University of Agriculture and Technology, 3-5-8 Saiwai, Fuchu-City, Tokyo 183-8509, Japan; 2 Advanced Clinical Research Center, Institute of Neurological Disorders, 255 Furusawa-Tsuko, Asao-ku, Kanagawa 215-0026, Japan; 3 Laboratory of Molecular Neurobiology, Graduate School of Biostudies, Kyoto University, Yoshidakonoe-cho, Sakyo-ku, Kyoto 606-8501, Japan; 4 Laboratory of Molecular Neurobiology, Graduate School of Pharmaceutical Sciences, Kyoto University, Yoshidakonoe-cho, Sakyo-ku, Kyoto 606-8501, Japan

**Keywords:** Oleoylethanolamide, GPR119, Satiety, Distal gastrointestinal tract, Gut microbiota, CCK, cholecystokinin, FAE, fatty acid ethanolamide, LPC, lysophosphatidylcholine, OA, oleic acid, OEA, oleoylethanolamide, GLP-1, glucagon-like peptide 1, GPCR, G protein-coupled receptor, GPR119, G protein-coupled receptor 119, MAG, monoacylglycerols, PPARα, peroxisome proliferator-activated receptor α, PYY, peptide YY

## Abstract

**Objective:**

The gastrointestinal tract affects physiological activities and behavior by secreting hormones and generating signals through the activation of nutrient sensors. GPR119, a lipid sensor, is indirectly involved in the secretion of incretins, such as glucagon-like peptide-1 and glucose-dependent insulinotropic peptide, by enteroendocrine cells, while it directly stimulates insulin secretion by pancreatic beta cells. Since GPR119 has the potential to modulate metabolic homeostasis in obesity and diabetes, it has attracted interest as a therapeutic target. However, previous studies have shown that the deletion of *Gpr119* in mice does not affect glucose homeostasis and appetite in either basal or high-fat diet-fed conditions. Therefore, the present study aimed to explore the role of GPR119 signaling system in energy metabolism and feeding behavior in mice.

**Methods:**

*Gpr119* knockout (KO) mice were generated using CRISPR-Cas9 gene-editing technology, and their feeding behavior and energy metabolism were evaluated and compared with those of wild type (WT) mice.

**Results:**

Upon inducing metabolic stress via food deprivation, *Gpr119* KO mice exhibited lower blood glucose levels and a higher body weight reduction compared to WT mice. Although food intake in WT and KO mice were similar under free-feeding conditions, *Gpr119* KO mice exhibited increased food intake when they were refed after 24 h of food deprivation. Further, food-deprived *Gpr119* KO mice presented shorter post-meal intervals and lower satiety for second and later meals during refeeding, resulting in increased food intake. Associated with this meal pattern, levels of oleoylethanolamide (OEA), an endogenous agonist of GPR119, in the luminal contents of the distal gastrointestinal tract were elevated within 2 h after refeeding. The large-intestinal infusion of OEA prolonged post-meal intervals and increased satiety in the first meal, but not the second meal. On the other hand, infusion of oleic acid increased cecal OEA levels at 2 h from the beginning of infusion, while prolonging post-meal intervals and increasing satiety on the meals that occurred approximately 2 h after the infusion. Cecal OEA levels were low in antibiotic-treated mice, suggesting that the gut microbiota partially synthesizes OEA from oleic acid.

**Conclusions:**

Collectively, our results indicate that the activation of gastrointestinal GPR119 by microbiota-produced OEA derived from oleic acid is associated with satiety control and energy homeostasis under energy shortage conditions.

## Highlights

• *Gpr119* is highly expressed in the distal intestine.
• GPR119 controls meal satiety and energy homeostasis under energy shortage conditions.
• OEA is synthesized from dietary oleic acid by gut microbiota.

## 1 Introduction

The gastrointestinal tract is involved in physiological regulation, including regulation of metabolism and feeding behavior, through the secretion of gut hormones and generation of signals via receptors in response to nutrients. Several G protein-coupled receptors (GPCRs) have been identified as sensors of lipids, such as fatty acids, monoacylglycerols (MAGs), and their metabolites, the levels of which are increased in the intestine after meals [1,2]. GPR40 and 120 are well-known receptors for dietary long-chain fatty acids and their metabolites produced by gut microbiota [1,3,4]. In addition, GPR119 is a receptor for MAGs [i.e. 2-oleoylglycerol (2-OG)], lysophosphatidylcholine (LPC), and fatty acid ethanolamides (FAEs) [i.e. oleoylethanolamide (OEA)] [5]. Although enterocytes, enteroendocrine cells, and neural fibers have been postulated to sense lipids via GPCRs in the gut, most studies imply that enteroendocrine cells are the primary cells that sense lipids, which results in the production of hormones like cholecystokinin (CCK) and glucagon-like peptide-1 (GLP-1) after a meal [6,7].

As indicated by previous *in vitro* and *in vivo* studies, GPR119 is highly expressed in the pancreas (pancreatic beta cells) and the gut, suggesting that GPR119 directly controls insulin secretion by the pancreas and indirectly controls incretin secretion by the intestine [5,8]. However, *Gpr119* knockout (KO) mice show no changes in islet morphology and size, glucose homeostasis, insulin levels, and appetite under either lab-chow- or high-fat diet-fed conditions [8,9]. In addition, endogenous ligands, such as LPC and OEA, have failed to enhance glucose-stimulated insulin secretion (GSIS) by islets isolated from either wild type (WT) or *Gpr119* KO mice [8]. Interestingly, EX-4, a GLP-1 receptor agonist, enhanced GSIS in both WT and *Gpr119* KO mice, while *Gpr119* KO mice exhibited reduced postprandial plasma GLP-1 levels [8]. Therefore, the physiological function of intestinal GPR119 as a pharmacological target for regulating metabolism and feeding behavior should be further elucidated. Notably, a recent study suggested that the activation of distal-intestinal GPR119 slowed gastric emptying and reduced food intake in *Cyp8b1* KO mice that exhibited impaired lipid absorption in the upper gut and absorbed dietary fat from the distal intestine [10].

OEA, an endogenous ligand of GPR119, is a well-known lipid mediator that modulates lipid metabolism and feeding behavior [11]. Detailed investigations have shown that OEA is directly derived from the diet or synthesized from dietary oleic acid (OA) in gastrointestinal enterocytes, especially in the proximal intestine [12,13]. OEA produced in the proximal intestine plays a vital role in inducing meal satiety by activating sensory fibers of the vagus nerve via peroxisome proliferator-activated receptor α (PPARα) activation [13,14]. In addition, a study suggests that OEA can modulate the secretion of the gastrointestinal peptide hormone GLP-1, which induces satiation, by enteroendocrine cells [15]. Therefore, GPR119 may be involved in modulating feeding behavior through hormone secretion when ligands are formed in the distal intestine; however, OEA levels in the distal-intestinal tissue are unaffected after meals [12]. Studies indicate that gut microbiota produce various types of small molecules, including *N*-oleoylserinol, which was recently identified as a GPR119 ligand (EC_50_, 1.6 μM), and FAE [[16], [17], [18]]. FAEs naturally exist as signaling molecules in many organisms, from simple life forms to humans. Therefore, intestinal GPR119 may be activated by biomolecules produced by the gut microbiota, such as *N*-oleoylserinol and FAEs. However, the effects of these molecules on the host via GPR119 activation have not been elucidated yet.

The present study investigated the role of intestinal GPR119 in feeding behavior and energy homeostasis using a new *Gpr119* KO mouse line. Our results indicate that GPR119 is essential for modulating energy homeostasis, particularly during energy deficit, and that the distal-intestinal GPR119 induces satiety in modulating meal patterns by sensing microbiota-produced metabolites such as OEA.

## 2 Material and methods

### 2.1 Animals

C57BL/6 J mice were obtained from Japan SLC Inc. (Tokyo, Japan). *Gpr119* KO mice were generated using the CRISPR-Cas9 genome editing system by Unitech Co., Ltd. (Kashiwa, Japan) (see details below). Mice were maintained with CLEA Rodent Diet CE-2 (CLEA Japan, Inc., Tokyo, Japan) and housed under the following conditions: room temperature (20–25 °C), 40–60% humidity, and a 12/12 h light–dark cycle (light period: 8:00–20:00). All animal experiments were performed in accordance with the guidelines of the Committee on the Ethics of Animal Experiments of Tokyo University of Agriculture and Technology. The Animal Research Ethics Subcommittee approved the experiments conducted by the Tokyo University of Agriculture and Technology (permit: 28–87).

### 2.2 *Gpr119* knockout mouse model

*Gpr119* KO mice were generated using the CRISPR-Cas9 genome editing system by consignment production (Unitech Co., Ltd.). gRNA sequences (target sequence 1, TCAGAGTCACAGCACCGTTC; target sequence 2, GTACAGGTATACTCGCTTCA) were designed with the following PAM sequence outside the protein coding sequence (CDS) of GPR119 to delete the whole CDS and gRNAs were microinjected into fertilized embryos of C57BL/6 J mice with Cas9 mRNA (Supplemental Fig. 1A). Four male mice (KO1–4) were obtained from Unitech Inc. after screening for the deletion of target sequences using a genotyping primer set (WT allele, 2199 bp; target allele, approximately 800 bp) (Table 1 and Supplemental Fig. 1C). One of the four mice (KO4) exhibited breeding issues. Hence, sequences of target alleles of rest of the mice (KO1–3) were analyzed by Sanger sequencing (Eurofins Genomics, K.K., Japan; Supplemental Fig. 1B). Finally, based on the sequence of the target allele and breeding ability of mice, KO1 was used as a *Gpr119* KO mice line in this study. Herein, F3–F6 litters were used. The confirmed sequence of the *Gpr119* KO mice (KO1) shows a 1372-bp deletion around the target and full deletion of the CDS of GPR119. Moreover, *Gpr119* was not detected in various tissues of *Gpr119* KO mice (Supplemental Fig. 1D; data for cecum and liver tissues are shown).

**Table 1:** Primers used for genotyping and qPCR.

Gene	Forward Primer Sequence (5′ −>3′)	Reverse Primer Sequence (5′ −>3′)
**qPCR**		
*18s*	CTCAACACGGGAAACCTCAC	AGACAAATCGCTCCACCAAC
*Gpr119*	TCCTCACCGTCATGCTGATTG	ATATGGAGACTCCGAGTGGGA
*Gcg*	ACAGAAGCGCATGAGGACC	TTGATGAAGTCCCTGGTGGC
*Pyy*	ACGGTCGCAATGCTGCTAAT	GCTGCGGGGACATCTCTTTTT
*Cck*	AGCGCGATACATCCAGCAG	ACGATGGGTATTCGTAGTCCTC
**Quantification of bacterial number**		
Total bacterial number	ACTCCTACGGGAGGCAGCAGT	ATTACCGCGGCTGCTGGC
**Genotyping**		
For *Gpr119* KO mice	ACGAAAAGTCCCTATCCTTTCATAG	AGAGAGGGATTGTTGTAGTCAATCA

### 2.3 Feeding restriction

For the experiments related to feeding restriction, mice were housed individually in wired-bottom cages to avoid coprophagia and were acclimated to the housing conditions for at least 3 days. Five different feeding conditions were set up for the respective groups: free-feeding (FF), food-deprived (FD), refeeding (RF)-1 h, RF-2 h, and RF-5 h. Mice in the FD group were deprived of food for 24 h (from 9:00 to 9:00). Mice in the RF-1 h, RF-2 h and RF-5 h groups were provided access to food for 1, 2, and 5 h, respectively, after 24 h food deprivation.

### 2.4 Antibiotic administration

Seven-week-old WT mice were given normal drinking water (control group) and drinking water containing ampicillin (0.5 mg/mL, sodium salt), neomycin (0.5 mg/mL, sulfate), metronidazole (0.5 mg/mL), and vancomycin (0.1 mg/mL) (antibiotic group). All the antibiotics were purchased from FUJIFILM Wako Pure Chemical Corporation (Tokyo, Japan). Meal patterns and food intake in the mice were observed after one week of antibiotic administration (with free access to water and food), and we collected the cecum to quantify lipids and total bacteria number in the mice. Total number of cecal bacteria was determined by real-time quantitative PCR (qPCR).

### 2.5 Energy metabolism and locomotor activity

Respiratory quotient, energy expenditure, and locomotor activity were evaluated in seven-week-old male mice. Mice were individually housed in a measurement chamber (LP-80CCFL-8AR; Nippon Medical & Chemical Instrument, Co., Ltd., Osaka, Japan) with free access to food and water and acclimatized for at least 12 h. VO_2_ and VCO_2_ levels were measured using the MK-5100MS device (Muromachi Kikai Co., Ltd., Tokyo, Japan), while locomotor activity was measured using the SUPERMEX PAT.P (Muromachi Kikai Co., Ltd.). The system was controlled by a 12 h light–dark cycle under atmospheric conditions of 22 °C and 30–60% humidity. Measurements were conducted for at least 48 h, including the acclimation period (≥12 h), and continuous 24-h raw data were obtained for calculations. Respiratory quotient and energy expenditure were calculated based on the VO_2_ and VCO_2_ values recorded using the MMS-4 software (version 6.2). The locomotor activity based on the counts of thermal radiation in mice was calculated using the CompACT AMS software (version 3; Muromachi Kikai Co., Ltd.). The EE ANCOVA analysis done for this work was provided by the 10.13039/100000062NIDDK Mouse Metabolic Phenotyping Centers (MMPC, www.mmpc.org) using their Energy Expenditure Analysis page (http://www.mmpc.org/shared/regression.aspx) and supported by grants DK076169 and DK115255. For ANCOVA analysis, energy expenditure raw data (kcal/day) were blotted against the individual body weight (g).

### 2.6 Large-intestinal catheter-attachment surgery

Catheters were surgically implanted at the end of the ileum to directly deliver lipids into the cecum. Both sides of the tubes (12 cm, 0.012″ ID × 0.037″ OD; Access Technologies, Skokie, IL, USA) were prefabricated to attach to a 3-mm square piece of monofilament polypropylene mesh approximately 2.0 cm from the end of the tube with silicon adhesive (3 M™ Clear Super Silicone Seal; 3 M, St. Paul, MN, USA). Mice were anesthetized with a mixture of medetomidine hydrochloride (0.3 mg/kg, i.p, Domitor®, Nippon Zenyaku Kogyo Co., Ltd., Fukushima, Japan), midazolam (4.0 mg/kg, i.p.; Dormicum®, Maruishi Pharmaceutical. Co., Ltd., Osaka, Japan), butorphanol tartrate (5.0 mg/kg, i.p.; Vetorphale®; Meiji Seika Pharma, Tokyo, Japan), and an incision (2.0 cm) was made through the skin and abdominal muscle along the midline of the lower abdomen. The lower ileum and cecum were gently exposed, and a small puncture in the ventral wall at approximately 1.0 cm proximal to the end of the ileum was made using a needle. One side of the tube (approximately 0.5 cm) was inserted through the puncture, facing the cecum. A tissue adhesive (Aron Alpha A Sankyo; Sankyo, Tokyo, Japan) was applied to the mesh for adherence to the gut wall. The outer portion of the tube with the attached square piece of mesh was subcutaneously routed to the back of the neck, exteriorized, and fixed to the underlying tissue by applying the tissue adhesive. The abdominal muscle wall was sutured using absorbent sutures [ELmelt; Natsume Seisakusho Co., Ltd., (Nastume), Tokyo, Japan], and the skin was closed using surgical sutures (silk; Natsume) or stainless-steel wound clips (AUTOCLIP®; Becton, Dickinson and Company, Franklin Lakes, NJ, USA). The outer end of the catheter was plugged with a stainless-steel pin to avoid leakage of intestinal fluid. After the operation, the mice were intraperitoneally injected with Antisedan® (3.0 mg/kg, atipamezole hydrochloride, Nippon Zenyaku Kogyo Co., Ltd.) for recovery against the action of medetomidine hydrochloride. The catheter was flushed with saline (0.5 mL) once every 2–3 days to avoid blockage. Following surgery, the mice were individually housed with free access to food and water and were allowed 7–10 days of surgical recovery before the initiation of experiments.

### 2.7 Lipid infusion from the large-intestinal catheter

The mice implanted with large-intestinal catheters were deprived of food for 24 h (from 9:00 to 9:00) before lipid infusion: OA (Sigma Aldrich, Burlington, MA, USA) or OEA (Cayman Chemical, Ann Arbor, MI, USA). OA was dissolved in phosphate-buffered saline (PBS) containing 0.3% xanthan gum (Tokyo Chemical Industry Co., Ltd., Tokyo, Japan) to obtain a final concentration of 10 mg/mL. OEA was dissolved in PBS containing 0.3% xanthan gum and 10% dimethyl sulfoxide (DMSO) to obtain a final concentration of 4 mg/mL. The infusion volume was 0.1 mL/mice, and infusion was manually conducted for 30 s. Mice were placed in a feeding measurement system right after lipid infusion. For lipid measurement in the cecum, mice were sacrificed to collect cecum samples 2 h after OA administration or 30 min after OEA administration.

### 2.8 Feeding behavior

Feeding activities of mice were recorded using a feeding measurement system MFI-01 equipped with an FIC-001 sensor (Muromachi Kikai Co., Ltd), which can monitor both access to the food container and weight of food in the food container. Raw data were collected every 5 min using the system with the CompACT AMS software. In addition, each test session was videotaped to verify the exact time of accessing the food container. Mice were acclimatized to the system and food containers for at least 3 days before the experiment. Meal pattern analysis was performed as described in a previous study by Gaetani et al. [19] with some modifications: meal interval (min), interval time between meals; minimal meal interval, 5 min; meal, feeding periods included bouts; meal latency (min), the time interval from the onset to the first eating episode; meal length (>1 min), time from start to finish of the meal; frequency (meals/session), meal number in the session period; total food intake (g/kg body weight/session), the amount of food consumed during the session; meal size (g/kg body weight), the amount of food consumed during the meal; satiety ratio [min/(g/kg)], the meal interval to the next meal per meal as an indicator of satisfaction.

### 2.9 Food intake measurement

Food intake was measured by manual weighing of food before and after a feeding period in several experiments that did not require the analysis of meal patterns. To test the effects of OEA on food intake under FF conditions, we intraperitoneally injected mice aged 11–16 weeks with OEA (20 mg/kg/mL) or a control vehicle (DMSO; Sigma Aldrich) at 19:30. The mice were then exposed to food at the onset of the dark period (20:00), and the remaining food was measured at the beginning of the light period (8:00). To test the effects of OEA on FD conditions, we intraperitoneally injected mice deprived of food for 24 h (10:00–10:00) with OEA (20 mg/kg/mL) or control vehicle (20% PBS in DMSO) 30 min before the start of the measurement (9:30). The mice were exposed to food for 5 h (10:00–15:00) to measure food intake.

### 2.10 Sample harvesting

At the end of the experiment, the mice were anesthetized using isoflurane (Pfizer Inc., New York, NY, USA), and tissue samples were collected. When needed, we measured blood glucose levels in the tail vein using a portable glucometer (OneTouch® Ultra®; LifeScan Inc., Milpitas, CA, USA) with an LFS quick sensor (LifeScan Inc.) before anesthesia was administered. Blood was collected from the portal vein and inferior vena cava using heparin-treated tubes (sodium salt; Yoshindo Inc., Toyama, Japan). The plasma was separated by centrifugation at 8,000×*g* for 5 min at 4 °C. The plasma samples were prepared for GLP-1 analysis by mixing a portion of blood obtained from the portal vein with dipeptidyl peptidase IV inhibitor (Merck Millipore, Burlington, MA, USA) at 1% (v/v). The gut (stomach, duodenum, jejunum, ileum, cecum, and colon) was opened, gut contents were collected if needed, and the tissue was rinsed with ice-cold PBS. Gut tissue samples and plasma were immediately frozen in chilled 2-methyl butane (Wako) on dry ice, and all samples were stored at −80 °C until further use. Fresh fecal droppings were collected during fasting for 5 h to analyze gut microbiota composition.

### 2.11 Lipid extraction

Lipid extraction was performed according to the method described in a previous study with minor modifications [20]. A sample of biological material (50 mg; intestinal tissue, intestinal contents, feces, diet) was added to 1 mL ice-cold methanol (Wako) containing internal standards [500 ng of 19:0 free fatty acid (FFA), Nu-chek Prep, Inc., Elysian, MN, USA; 500 ng of 19:0 MAG, Nu-chek Prep, Inc.; 10 pmol of d^4^-OEA, Cayman, Ann Arbor, MI, USA], and then homogenized. The total lipids were extracted by adding 2 mL of chloroform (Wako) and 0.75 mL of 0.5 M KCl to the homogenate. After mixing and centrifugation, the separated lower layer (organic layer) was dried under nitrogen gas, and the residue was reconstituted by adding 100 μL of solvent mixture [chloroform: methanol, 1:3 (v/v)] for LC**–**MS/MS analysis. To assess triacylglycerols in the diet, we extracted the total lipids as stated above without an internal standard, and the residue was dissolved in 50 μL of 2-propanol (Wako).

### 2.12 Quantification of lipids by LC–MS/MS

FAEs, MAGs and FFAs were quantified using a mass spectrometer (Xevo TQD; Waters, Milford, MA, USA) coupled with the Acquity UPLC system (Waters). For FAE analysis, ZORBAX Eclipse XDB C18 column (2.1 mm × 100 mm inner diameter, 1.8 μm; Agilent Technologies, Santa Clara, CA, USA) was used for separation using an acetonitrile gradient. Solvent A was 0.1% formic acid (Wako), and solvent B was acetonitrile (Wako). The gradient was applied as follows: 0–10 min, 70% B; 10–20 min, 70–100% B linear gradient; 20–25 min, 100% B; 25–25.1 min, 100–70% B linear gradient; and 25.1–30 min, 70% B. The flow rate was 0.3 mL/min, and the column temperature was maintained at 20 °C. The electrospray ionization interface remained in the positive ionization mode, and the ion spray voltage was set to −3000 V. For FFA analysis, an Acquity UPLC BEH C18 column (1.0 mm × 150 mm × 1.7 μm; Waters) was used for separation using a methanol gradient. Solvent A was 10 mM ammonium acetate (Wako), and solvent B was methanol (Wako). The gradient was applied as follows: 0–1 min, 90–100% B linear gradient; 1–6 min, 100% B; 6–6.2 min, 100–90% B linear gradient; and 6.2–9 min, 90%. The flow rate was 0.3 mL/min, and the column temperature was maintained at 50 °C. The electrospray ionization interface remained in the negative ionization mode, and the ion spray voltage was set to 2500 V. For MAG analysis, an Acquity UPLC BEH C18 column was used for separation using a methanol gradient. Solvent A was 0.1% formic acid, and solvent B was methanol. The gradient was applied as follows: 0–1 min, 90–100% B linear gradient; 1–6 min, 100% B; 6–6.2 min, 100–90% B linear gradient; and 6.2–9 min, 90%. The flow rate was 0.3 mL/min, and the column temperature was maintained at 50 °C. The electrospray ionization interface remained in the positive ionization mode, and the ion spray voltage was set to −3000 V. For those analyses, nitrogen gas was used as the drying gas at a flow rate of 15 L/min and as the nebulizer gas at a flow rate of 3 L/min. The desolvation and heat block temperatures were set to 250 °C and 400 °C, respectively. Argon at a pressure of 230 kPa was used as the collision-induced dissociation gas. The temperature in the autosampler was maintained at 15 °C. Individually optimized multiple reaction monitoring (MRM) parameters were determined for each transition for both target compounds and internal standards. A dwelling time of 100 ms was used for all the MRM transitions. The collision energies were −15 V. MRM was used to quantify the FAEs (*m*/*z* 326.5 > 62.2 for OEA and *m*/*z* 330.5 > 66.2 for d^4^-OEA), MAGs (*m*/*z* 357.5 > 265.5 for 2-OG and *m*/*z* 373.5 > 281.5 for 19:0 MAG) and FFAs (*m*/*z* 281.5 > 281.5 for 18:1 and *m*/*z* 297.5 > 297.5 for 19:0). Peaks were identified based on the retention time, and only those peaks with a signal to noise (S/N) ratio of 10 or more were used for the calculation. The absolute levels of OEA, 2-OG and 18:1 FFA were quantified using standard curves.

### 2.13 mRNA extraction and qPCR analysis

Total RNA from animal tissue was extracted using the RNAiso Plus reagent (Takara Bio Inc., Shiga, Japan) and further purified using the RNeasy Mini kit (QIAGEN, Venlo, Netherlands) with DNase. First-strand complementary DNA (cDNA) was synthesized from 500 ng of RNA by reverse transcription using SuperScript III™ Reverse Transcriptase (Thermo Fisher Scientific, Waltham, MA, USA). qPCR was performed using the StepOne™ Real-Time PCR System (Thermo Fisher Scientific) with SYBR® Premix Ex Taq™ II (Takara). The primer sequences used are listed in Table 1. The expression of each gene was analyzed according to the comparative cycle threshold method. The mRNA levels of the target genes were normalized to the mRNA levels of 18S rRNA as the housekeeping gene.

### 2.14 Analysis of gut microbiota by 16S rRNA gene sequencing and qPCR

Total DNA was extracted from frozen feces and cecal contents using the Fast DNA™ Spin kit (MP Biomedicals, LLC., Irvine, CA, USA) according to the manufacturer's instructions. The V3–V4 region of the 16S rRNA gene was amplified using the following primers: forward (5′-TCGTCGGCAGCGTCAGATGTGTATAAGAGACAGCCTACGGGNGGCWGCAG-3′) and reverse (5′-GTCTCGTGGGCTCGGAGATGTGTATAAGAGACAGGACTACHVGGGTATCTAATCC-3′).

The amplicon was purified using AMPure XP reagent (Beckman Coulter, Brea, CA, USA), and DNA sample quality was determined using the Bioanalyzer High Sensitivity DNA Analysis kit (Agilent Technologies). The 16S rRNA sequence was determined using the Illumina MiSeq system with a MiSeq Reagent 222 kit v3 (Illumina, San Diego, CA, USA) using purified libraries according to the “Preparing Libraries for Sequencing on the MiSeq” (part 15039740, Rev. D) protocol. The 16S microbial sequencing data were processed using Quantitative Insights into Microbial Ecology software (version 1.9.1; http://www.quiime.org). Samples with less than 100,000 reads were excluded from further analysis. The raw sequences were aligned using the Python Nearest Alignment Space Termination tool with the SILVA core-set alignment template (Silva version 128) to obtain bacterial operational taxonomic units (OTUs). The raw OTU data (relative abundance) were filtered by domain (bacteria only) and excluded based on the average abundance of each bacterium (<0.0001 for phylum and <0.00001 for family), which were then re-normalized. Raw data were deposited into the DNA Data Bank of Japan database (Accession No. DRA014241). In addition, total bacterial number in the cecum of mice was determined using qPCR (see details above) [21]. Primer sequences are shown in Table 1, and DNA obtained from *Bifidobacterium breve* (JCM1192T; Japan Collection of Microorganisms, RIKEN BioResource Research Center, Tsukuba, Japan) was used as standard for quantification.

### 2.15 Histological analysis

Fresh cecum was washed with PBS, embedded in Tissue-Tek® OCT compound (Sakura Finetek Japan Co. Ltd., Tokyo, Japan) and frozen at −80 °C until freeze sectioning. We prepared 10 μm-sections using a cryostat (Leica CM1860, Leica Biosystems, Wetzlar, Germany) at −20 °C, and the sections were fixed on glass slides with 4% paraformaldehyde for 10 min for hematoxylin-eosin (HE) staining. Briefly, the sections were immersed in hematoxylin solution and placed in 70% ethanol for color fixation. Next, the sections were immersed in eosin solution for 4 min. After washing with running water, we immersed the sections in 70% ethanol, twice in 100% ethanol for dehydration, and twice in xylene to make the sections transparent. After air-drying, the sections were sealed with Mount-Quick (Daido Sangyo, Toda, Japan) and cover glass. After 1 h, the sections were observed under a microscope (BZ-X710, Keyence Co., Osaka, Japan). At least two sections of cecum tissue obtained from each mouse were selected for determining villi length (μm) and number of villi (number/μm of serosa length in transverse section) using ImageJ software (NIH, Bethesda, MD, USA).

### 2.16 Cell culture and cAMP assay

Ligand activity of OEA and 2-OG on GPR119 was assessed using HEK-293 cells expressing doxycycline (DOX)-inducible human GPR119 following by previous reports [3,21]. Briefly, Flp-In T-REx HEK293 cells (Invitrogen, Thermo Fisher Scientific) were transfected with a mixture of pcDNA5/FRT/TO- FLAG-hGPR119 and pOG44 using the Lipofectamine reagent (Invitrogen, Thermo Fisher Scientific) [21,22]. The cells were maintained in Dulbecco's modified Eagle's medium (DMEM, high-glucose, Sigma-Aldrich) containing 10 μg/mL blasticidin S (Cayman), 100 μg/mL hygromycin B (Gibco, Grand Island, NY, USA), and 10% fetal bovine serum (Gibco). Cells were cultured at 37 °C and 5% CO_2_. For cAMP determination, the cells were seeded in 24-well plates (2 × 10^5^ cells/mL/well), cultured for 24 h, and treated with or without DOX (10 μg/mL, Wako) for 24 h. The cells were pre-treated with 3-isobutyl 1-methylxanthine (500 μM, Sigma) for 30 min, and then stimulated with forskolin (2 μM, Sigma), Ar231453 (0.3 μM and 3 μM, Sigma), OEA (30 μM) and 2-OG (30 μM) for 10 min. The cAMP levels were determined using LC–MS/MS.

### 2.17 Other analysis

Plasma GLP-1 concentrations were measured using the GLP-1 (Active) ELISA kit (Merck Millipore) according to the manufacturer's protocol. Plasma FFA concentrations were measured using the LabAssay™ NEFA laboratory assay kit (Wako). Total plasma cholesterol concentrations were measured using the LabAssay™ Cholesterol kit (Wako). Plasma triglyceride concentrations were measured using the LabAssay™ triglycerides kit (Wako).

### 2.18 Statistical analysis

The measured values are expressed as mean ± standard error of mean (SEM). Statistically significant differences between groups were analyzed using the following tests: two-group comparison, Student's *t*-test; three-group comparison, one-way analysis of variance (ANOVA) and Tukey's multiple comparisons test; and two-group correspondence test, two-way ANOVA and Bonferroni's multiple comparisons test. ∗ Represents *p* < 0.05, ∗∗ represents *p* < 0.01, and ∗∗∗ represents *p* < 0.001.

## 3 Results

### 3.1 *Gpr119* KO mice exhibit reduced body weight and increased energy expenditure

**Figure 1:**
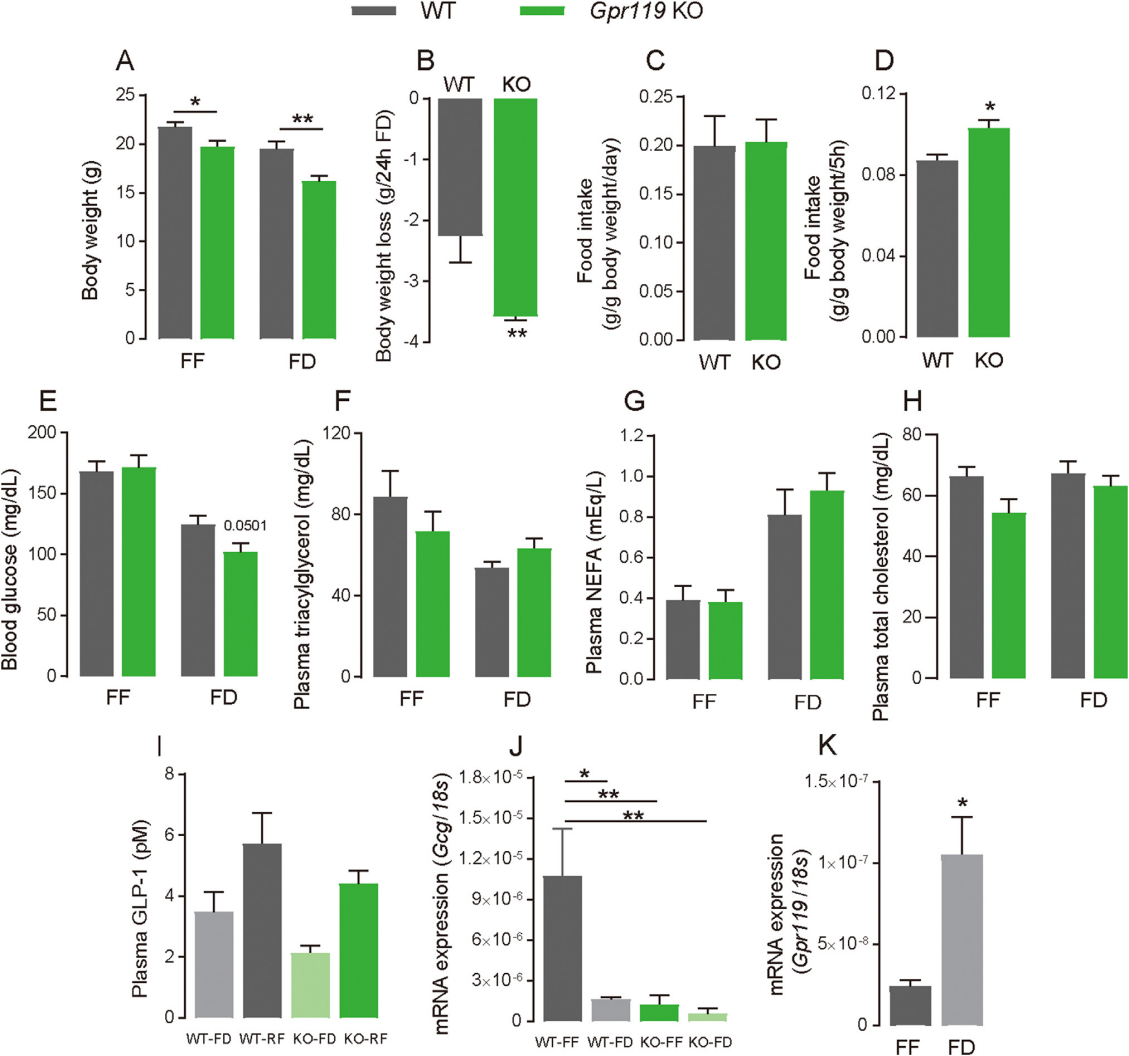
**Body weight, food intake, blood glucose, plasma lipids and GLP-1 in *Gpr119* KO mice under either free-feeding (FF) or food-deprived (FD) conditions**. (A) Body weight (n = 9), (E) blood glucose (n = 5), (F) plasma triacylglycerol (n = 8 for WT and n = 10 for KO), (G) non-esterified fatty acid (NEFA) (n = 8 for WT and n = 10 for KO), and (H) total cholesterol (n = 8 for WT and n = 10 for KO) were measured in seven-week-old WT and *Gpr119* KO mice under FF or 24-h FD conditions. (B) Body weight loss was calculated using individual body weight values as shown in (A). (C) Food intake over 24 h in WT or *Gpr119* KO mice under FF conditions (n = 5 for WT and n = 7 for KO). (D) Food intake during the 5-h refeeding period of mice after 24 h food deprivation (n = 5 for WT and n = 7 for KO). (I) Plasma GLP-1 in WT and *Gpr119* KO mice under FF and re-feeding (RF) conditions (n = 4 for FD and n = 5 for RF). (J) *Gcg* expressions in the cecum tissue of WT and *Gpr119* KO mice under FF and FD conditions (n = 4 for WT-FF, WT-FD, and KO-FD and n = 5 for KO-FF). (K) *Gpr119* expressions in the cecal tissue of WT mice under FF and FD conditions (n = 4). Statistical significance is indicated by asterisks (∗*p* < 0.05 and ∗∗*p* < 0.01). WT, wild type; KO, knockout.

**Figure 2:**
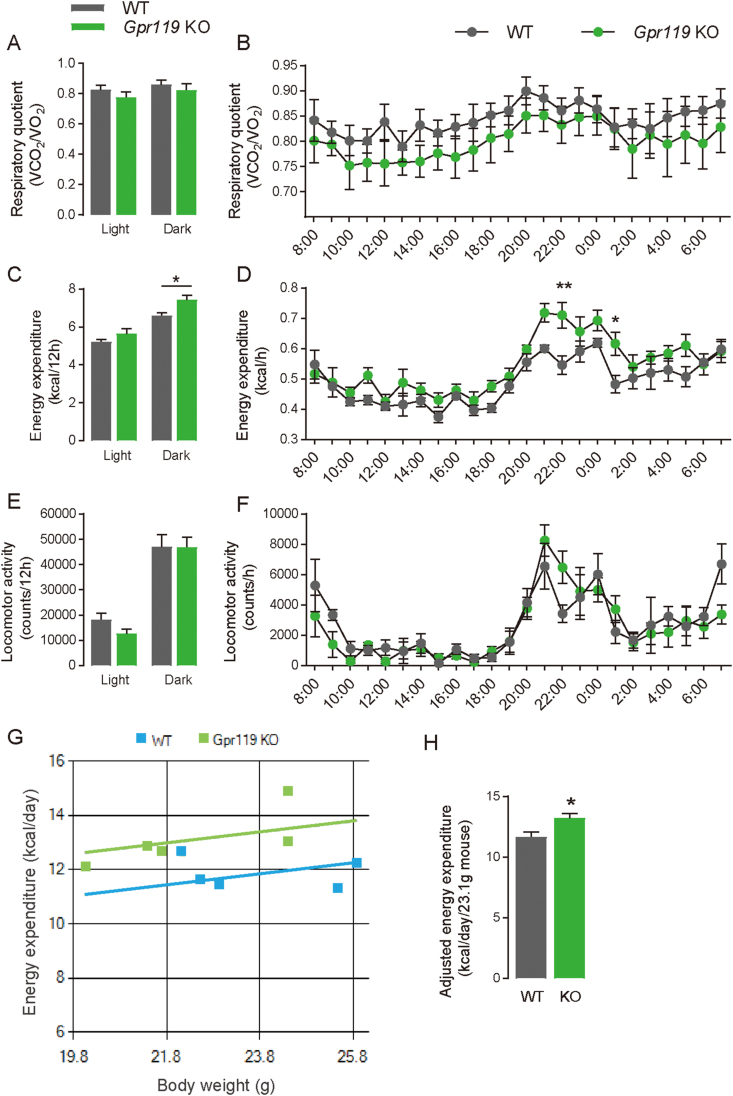
**Metabolic analysis of WT and *Gpr119* KO mice under FF conditions**. Metabolic parameters were analyzed in seven-week-old WT and *Gpr119* KO mice using a measurement device (MK-5100MS) (n = 5). Respiratory quotient, energy expenditure, and locomotor activity were calculated in the mice under FF conditions. Average of (A) respiratory quotient, (C) energy expenditure, and (E) locomotor activity during the dark and light cycles. Hourly changes of (B) respiratory quotient, (D) energy expenditure, and (F) locomotor activity over 24 h. The MMPC Energy Expenditure analysis was performed using raw data of body weight and energy expenditure of WT and *Gpr119* KO mice. (G) Individual energy expenditure plotted against corresponding body weight in mice. (H) ANCOVA predicted energy expenditure at a mean body weight of 23.1 g in seven-week old WT and *Gpr119* KO mice. Data are expressed as means ± SEM. Statistical significance is indicated by asterisks (∗*p* < 0.05 and ∗∗*p* < 0.01).

*Gpr119* KO mice were generated using the CRISPR-Cas9 genome-editing system to evaluate the physiological roles of GPR119. As shown in Supplementary Fig. 1, a 1372 bp-deletion around the target gene was confirmed by Sanger sequencing analysis. *Gpr119* KO mice maintained under FF conditions exhibited lower body weight (Figure 1A, *p* = 0.012) than that exhibited by littermate WT mice. However, no differences in growth, food intake (Figure 1C), and blood glucose levels (Figure 1E) were observed between WT and *Gpr119* KO mice maintained under FF conditions. Furthermore, no differences in plasma triacylglycerol (TG; Figure 1F), non-esterified fatty acid (NEFA; Figure 1G), and total cholesterol (Figure 1H) levels were observed between WT and *Gpr119* KO mice maintained under FF conditions. Therefore, energy metabolism and locomotor activity were evaluated in these mice. Energy expenditure (Figure 2C, *p* = 0.0146 for the dark cycle; Figure 2D, *p* = 0.003 for 22:00, *p* = 0.0423 for 1:00), but not respiratory quotient (Figure 2A,B) and locomotor activity (Figure 2E,F), was higher in the *Gpr119* KO mice than that in the littermate WT. Thus, *Gpr119* KO mice expended more energy than that expended by WT mice even when consuming similar amounts of food. Further, we assessed the MMPC Energy Expenditure analysis using raw data of bodyweight and energy expenditure to examine if increased energy expenditure mainly impacts body weight. The ANCOVA genotype effect considering bodyweight as covariate was not significantly different (Figure 2G, *p* = 0.126). Adjusted energy expenditure (kcal/day/23.1 g mouse) was significantly increased in *Gpr119* KO mice compared with WT mice (Figure 2H, *p* = 0.0276), which explains the relatively lean phenotype of *Gpr119* KO mice compared with WT mice.

### 3.2 *Gpr119* KO mice display hyperphagic responses during RF after food deprivation

To investigate the role of GPR119 in metabolic stress, we subjected the seven-week-old *Gpr119* KO mice and WT littermates to food-deprivation for 24 h. *Gpr119* KO mice showed lower body weight than WT mice under FD conditions (Figure 1A, *p* = 0.0024). The reduction in body weight in *Gpr119* KO mice was significantly greater than that in littermate WT mice (Figure 1B, *p* = 0.0078). Interestingly, *Gpr119* KO mice consumed more food than that consumed by WT mice 5 h after 24 h of food deprivation (Figure 1D, *p* = 0.049). In addition, blood glucose levels tended to be lower in *Gpr119* KO mice than in WT mice (Figure 1E, *p* = 0.0501). No differences in plasma TG (Figure 1F), NEFA (Figure 1G), and total cholesterol (Figure 1H) levels were observed between *Gpr119* KO and WT mice deprived of food for 24 h. These results indicate that *Gpr119* KO mice exhibited hyperphagic responses under the stress of food deprivation.

### 3.3 *Gpr119* KO mice exhibit shorter post-meal intervals during RF after food deprivation

**Figure 3:**
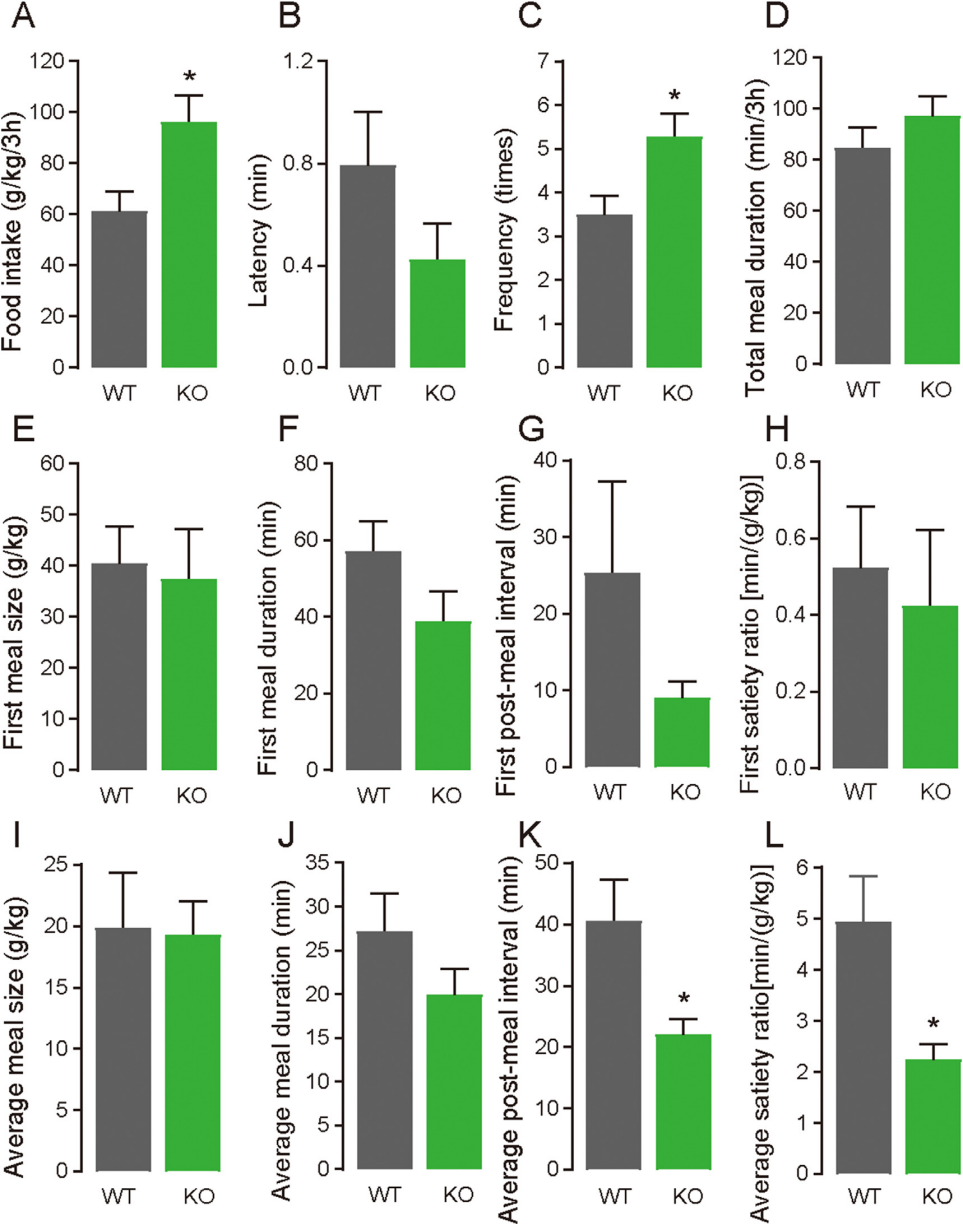
***Gpr119* KO mice exhibited increased food intake and reduced satiety for subsequent meals under re-feeding conditions**. After 24 h of food deprivation, WT or *Gpr119* KO mice were individually placed in a cage with food in the feeding behavior analysis system, and their behavior was recorded for up to 5 h. The following feeding parameters were analyzed in the first 3 h: (A) food intake, (B) latency, (C) meal frequency, (D) total meal duration, (E) first meal size, (F) first meal duration, (G) first post-meal interval, (H) first satiety ratio, (I) average meal size, (J) average meal duration, (K) average post-meal interval, and (L) average satiety ratio. Data are expressed as means ± SEM (n = 6 for WT and n = 7 for KO; n = 5 for latency of KO). Statistical significance is indicated by asterisks (∗*p* < 0.05). WT, wild type; KO, knockout.

Following the results in Figure 1 regarding the hyperphagic response of FD *Gpr119* KO mice, meal patterns were observed during 3 h of RF after 24 h of food deprivation in both WT and *Gpr119* KO mice using a feeding measurement system (Figure 3). Consistent with the results of the manual measurement of food intake shown in Figure 1D (RF-5 h), food intake measured using the system was significantly increased in *Gpr119* KO mice compared with that in WT mice (Figure 3A, *p* = 0.0201). Furthermore, meal frequency (Figure 3C) was significantly higher in *Gpr119* KO mice than in WT mice, while no differences in latency (Figure 3B) or total meal duration (Figure 3D) in the session were observed between the *Gpr119* KO and WT mice. No differences in the first meal parameters, such as meal size (Figure 3E), meal duration (Figure 3F), post-meal interval (Figure 3G), and satiety ratio (Figure 3H) were observed between the *Gpr119* KO and WT mice. Interestingly, the average post-meal interval (Figure 3K) and average satiety ratio (Figure 3L), but not average meal size (Figure 3I) and average duration (Figure 3J), were significantly decreased in *Gpr119* KO mice compared with those in WT mice. Therefore, we analyzed second meal parameters, results of which indicated that post-meal interval (Supplemental Fig. 2C, *p* = 0.065) and satiety ratio (Supplemental Fig. 2D, *p* = 0.0077) of the second meal were lower in *Gpr119* KO mice than in WT mice. No differences in the second meal size (Supplemental Fig. 2A) and meal duration (Supplemental Fig. 2B) were observed between the *Gpr119* KO and WT mice. These results suggested that *Gpr119* deletion induced a reduction in meal satiety during RF after food deprivation, and its effect was specifically delayed from the onset of feeding.

Further, we measured plasma GLP-1 levels in WT and *Gpr119* KO mice under FD and RF conditions (Figure 1I). GLP-1 levels showed an increasing tendency under RF in WT and *Gpr119* KO mice; however, there was no statistical difference between feeding conditions. In addition, deletion of *Gpr119* did not impact plasma GLP-1 levels. In addition, we verified the effects of food deprivation on *Gcg* and *Gpr119* expressions in the cecum. Interestingly, *Gpr119* expression was increased by food deprivation in the cecum of WT mice (Figure 1K, *p* = 0.0128), while *Gcg* expression was decreased (Figure 1J, *p* = 0.0119). In *Gpr119* KO mice, *Gcg* expression was not affected by the feeding conditions; however, the expression levels were relatively low compared to WT-FF (Figure 1J, *p* = 0.0062, WT-FF v.s. KO-FF; p = 0.0054, WT-FF vs KO-FD).

### 3.4 *Gpr119* is not involved in satiety induced by systemic OEA

**Figure 4:**
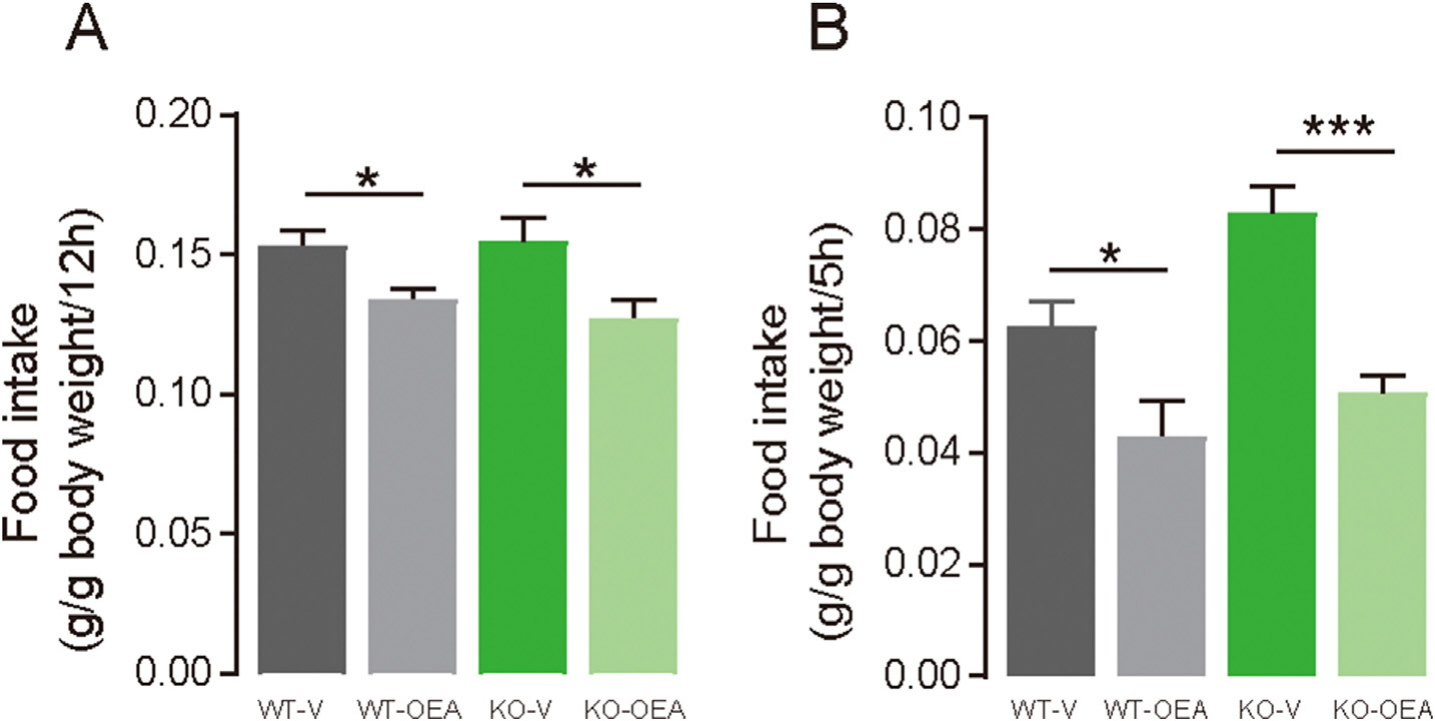
**Intraperitoneally injected OEA reduces food intake in both WT and *Gpr119* KO mice**. (A) Mice aged 11–16 weeks were intraperitoneally injected with OEA (20 mg/kg/mL) or control vehicle (DMSO) 30 min before the dark period. Then, food intake was measured in the dark period (20:00–8:00) (n = 7 for control vehicle, n = 6 for WT-OEA, and n = 8 for KO-OEA). (B) Mice were deprived of food for 24 h (10:00–10:00). Subsequently, they were intraperitoneally injected with OEA (20 mg/kg/mL) or control vehicle (20% PBS in DMSO) 30 min before the start of food intake measurement (9:30). Then, food intake was measured for 5 h (10:00–15:00; n = 5). Data are expressed as means ± SEM. Statistical significance is indicated by asterisks (∗*p* < 0.05 and ∗∗∗*p* < 0.001). WT, wild type; KO, knockout.

To investigate if GPR119 is involved in satiety induction by systemic treatment with OEA, food intake was measured in *Gpr119* KO mice that were intraperitoneally administered with OEA under FF or FD conditions. Under FF conditions, food intake in the dark cycle decreased as a result of OEA treatment in both WT (*p* = 0.016; Figure 4A) and *Gpr119* KO mice (*p* = 0.0203; Figure 4A) compared with that in mice that were treated with the vehicle. Consistent with Figure 1D, higher food intake was observed during 5 h of RF after food deprivation in *Gpr119* KO mice compared with that in WT mice under vehicle treatment; however, OEA treatment reduced food intake in both WT (*p* = 0.036; Figure 4B) and *Gpr119* KO mice (*p* < 0.001; Figure 4B). Overall, these results indicate that the induction of satiety by systemic OEA does not require GPR119 activation under either FF or FD conditions.

### 3.5 Feeding mobilizes OEA in intestinal contents

**Figure 5:**
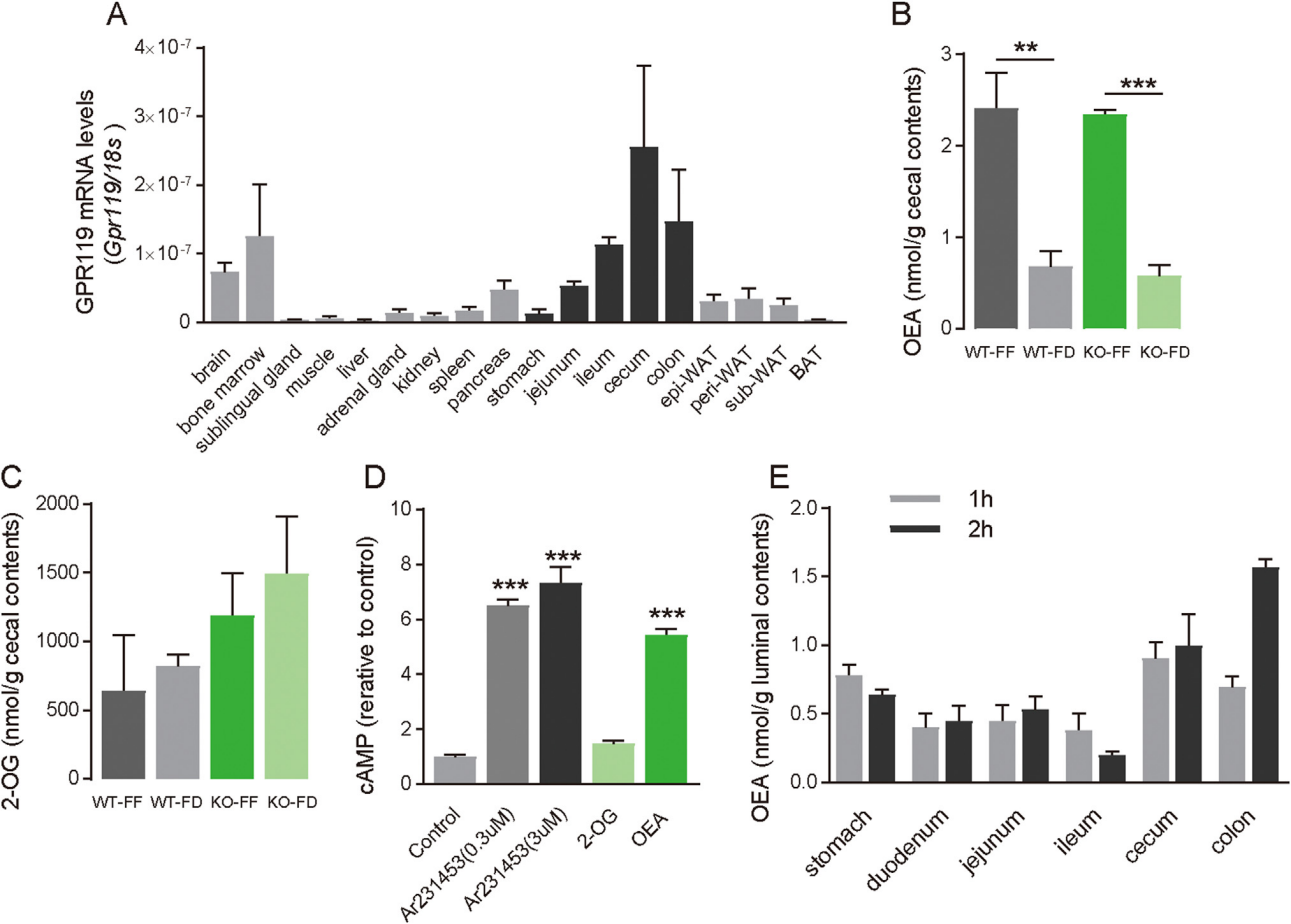
**Feeding mobilized OEA in the luminal contents of the distal intestine where *Gpr119* is highly expressed**. (A) GPR119 mRNA levels were measured in various tissue samples of 5 h-fasted seven-week-old WT mice. WAT, white adipose tissue. Epi, epididymal adipose tissue. Peri, perirenal adipose tissue. Sub, subcutaneous adipose tissue. BAT, brown adipose tissue (n = 3–4). (B) OEA and (C) 2-OG levels in cecal contents of mice under free-feeding (FF) or 24-h food-deprived (FD) conditions (n = 3 for WT-FF, KO-FF and KO-FD, n = 4 for WT-FD). (D) cAMP levels in human GPR119-expressing HEK293 cells treated with Ar231453, 2-OG, and OEA (n = 3). (E) OEA levels in intestinal contents of refed WT mice. Mice were deprived of food for 24 h and then refed for either 1 h or 2 h before the samples were harvested (n = 3 for 1 h samples, n = 3–4 for 2 h samples). Data are expressed as means ± SEM. Statistical significance is indicated by asterisks (∗∗*p* < 0.01, and ∗∗∗*p* < 0.001). WT, wild type; KO, knockout.

Here, we confirmed that *Gpr119* was highly expressed in the lower part of the gastrointestinal tract, including the ileum, cecum, and colon (Figure 5A). This expression pattern was associated with the expression of *Gcg* (Supplemental Fig. 3B) and *Pyy* (Supplemental Fig. 3C), but not *Cck* (Supplemental Fig. 3A) [23]. Analysis of the intestinal tissue of WT mice housed under FF conditions revealed that OEA levels were approximately 60–300 pmol/g tissue (Supplemental Fig. 3D). Therefore, we quantified OEA in the cecal contents of the mice where *Gpr119* was highly expressed; OEA levels in the cecal contents were 2.4 ± 0.4 nmol/g and 2.3 ± 0.0 nmol/g in WT and *Gpr119* KO mice, respectively, housed under FF conditions, and the levels decreased after 24-h food deprivation in WT (*p* = 0.0061; Figure 5B) and *Gpr119* KO mice (*p* < 0.001; Figure 5B). 2-OG levels in the cecum contents were similar between WT and *Gpr119* KO mice under FF conditions, and food deprivation did not affect cecal 2-OG levels in either WT and *Gpr119* KO mice (Figure 5C). Plasma OEA levels were similar between WT and *Gpr119* KO mice under FF conditions, and the levels were approximately 4 pmol/mL plasma (Supplemental Fig. 3E). In addition, *Gpr119* KO mice did not exhibit abnormal changes in the cecum tissues compared with WT mice, as determined by hematoxylin and eosin staining, and no changes in villi number and length (Supplemental Fig. 3F). Furthermore, we analyzed the microbiota composition of the fecal contents of WT and *Gpr119* KO mice. There was no significant difference at the phylum and family levels (Supplemental Fig. 3G) between WT and *Gpr119* KO mice.

Then, we tested ligand activity for GPR119 of OEA and 2-OG using HEK293 cells expressing DOX-inducible human GPR119. A synthetic agonist of GPR119, Ar231453 (0.3 μM and 3 μM), increased cellular cAMP levels by 7 times compared with vehicle control (Figure 5D), and Forskolin treatment increased cAMP levels by 27 times (data not shown). In the cells, OEA (30 μM) increased cAMP levels, whereas such increase was not observed with 2-OG treatment (30 μM). In DOX-uninduced cells, the activation by Ar231453 and OEA was not observed (data not shown). Therefore, we focused on OEA in the subsequent experiments.

The diet used in this study contained OEA at a concentration of 1.3 ± 0.2 nmol/g, which is much higher than the OEA levels in the gut tissue and plasma of the mice. Therefore, we expected that RF would alter the luminal levels of OEA because the diet contained OEA. Our analysis revealed that luminal OEA levels (nmol/g luminal contents) were higher in the cecum and colon than those in the duodenum, jejunum, and ileum in mice refed for 1 or 2 h (Figure 5E). However, cecal OEA levels in the mice refed for 2 h after food deprivation were not as high as those in the free-fed mice (Figure 5B). In addition, luminal OEA levels were slightly higher in the stomach than in the proximal intestine, suggesting dilution and absorption of OEA in the proximal intestine. These results suggest that luminal OEA levels in the large intestine were elevated as a result of feeding, which might cause the accumulation of dietary OEA or the production of OEA by gut microbiota [16,17].

### 3.6 Large-intestinal OEA infusion modifies meal patterns

**Figure 6:**
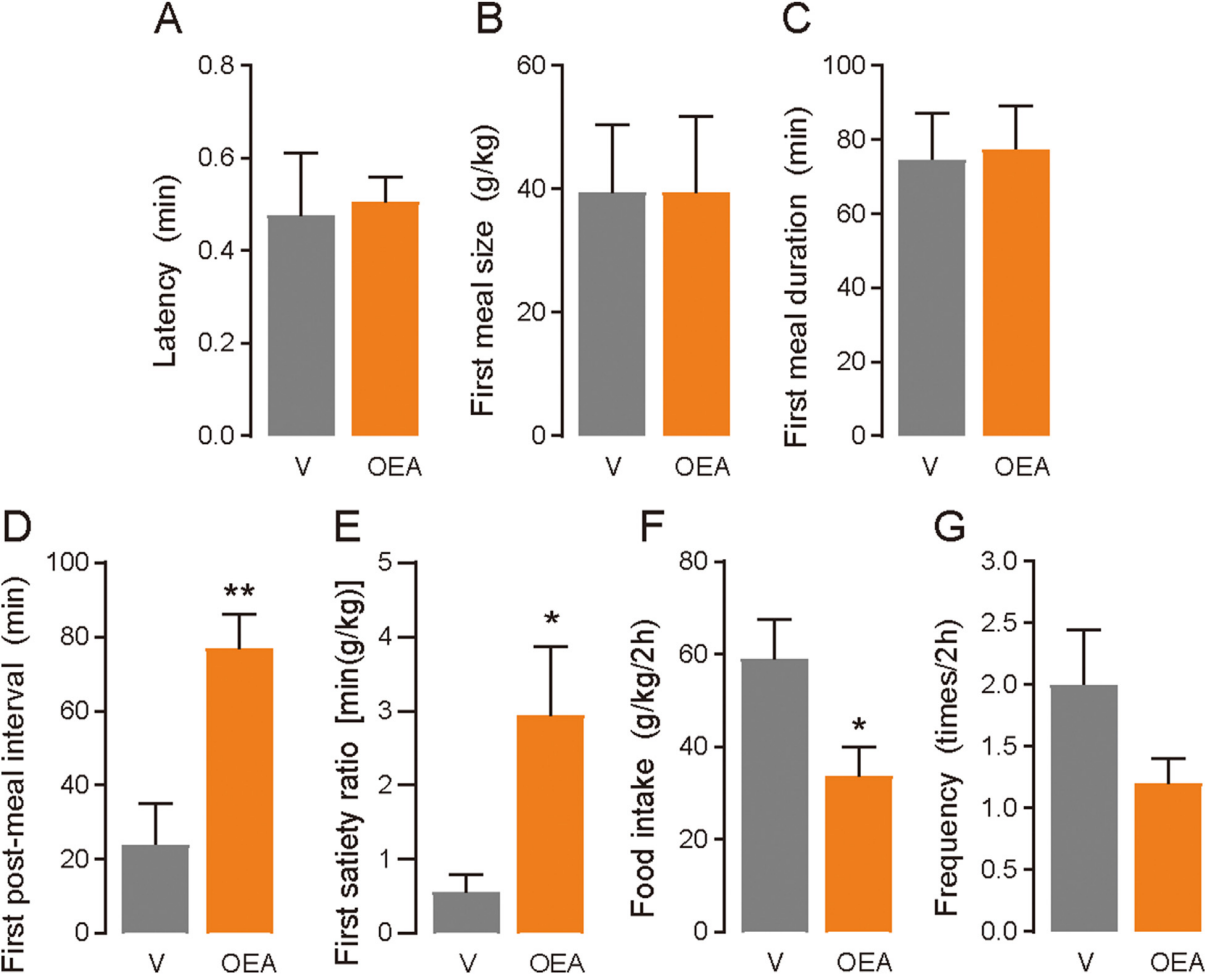
**Large-intestinal OEA infusion induced meal satiety in the mice**. Mice implanted with a catheter at the end of the ileum were deprived of food for 24 h before infusions. Infusions of OEA [0.4 mg/mice, OEA in phosphate-buffered saline (PBS) containing 0.3% xanthan gum and 10% DMSO] or vehicle (V; PBS containing 0.3% xanthan gum and 10% DMSO) were conducted for 30 s. The mice were placed into the feeding measurement system, and test sessions were recorded. Meal parameters were calculated using raw data, including (A) latency of feeding onset at the beginning of the trial, (B) first meal size, (C) first meal duration, (D) first post-meal interval, (E) first satiety ratio, (F) food intake, and (G) meal frequency (n = 5). Data are expressed as means ± SEM. Statistical significance is indicated by asterisks (∗*p* < 0.05, and ∗∗*p* < 0.01).

Next, we examined whether OEA infusion into the large intestine modified the meal pattern exhibited by mice because OEA was increased in the large intestine, where GPR119 was highly expressed (Figure 5). OEA was infused through a catheter inserted at the end of ileum after 24 h of food deprivation, and the meal behavior was monitored. In the OEA group, the first post-meal interval was significantly longer than that in the vehicle group (*p* = 0.0062; Figure 6D). In contrast, there were no differences in latency (Figure 6A), first meal size (Figure 6B), or first meal duration (Figure 6C) between the groups, resulting in an increased satiety ratio for the first meal in the OEA-infused mice (*p* = 0.0317; Figure 6E). Furthermore, we analyzed the meal pattern in 2 h of the mice, which showed that the total food intake (*p* = 0.0436; Figure 6F) and meal frequency (*p* = 0.100; Figure 6G) were lower in the OEA-infused mice. In addition, there were no differences in the average meal size, meal duration, post-meal interval, and satiety ratio between the groups (Supplemental Fig. 4), indicating that large-intestinal OEA infusion immediately affected the meal pattern; however, it was not long-lasting. These results collectively suggest that satiety can be induced by the sensing of OEA at the large intestine.

### 3.7 OEA generated from OA by gut microbiota modulates meal behavior

**Figure 7:**
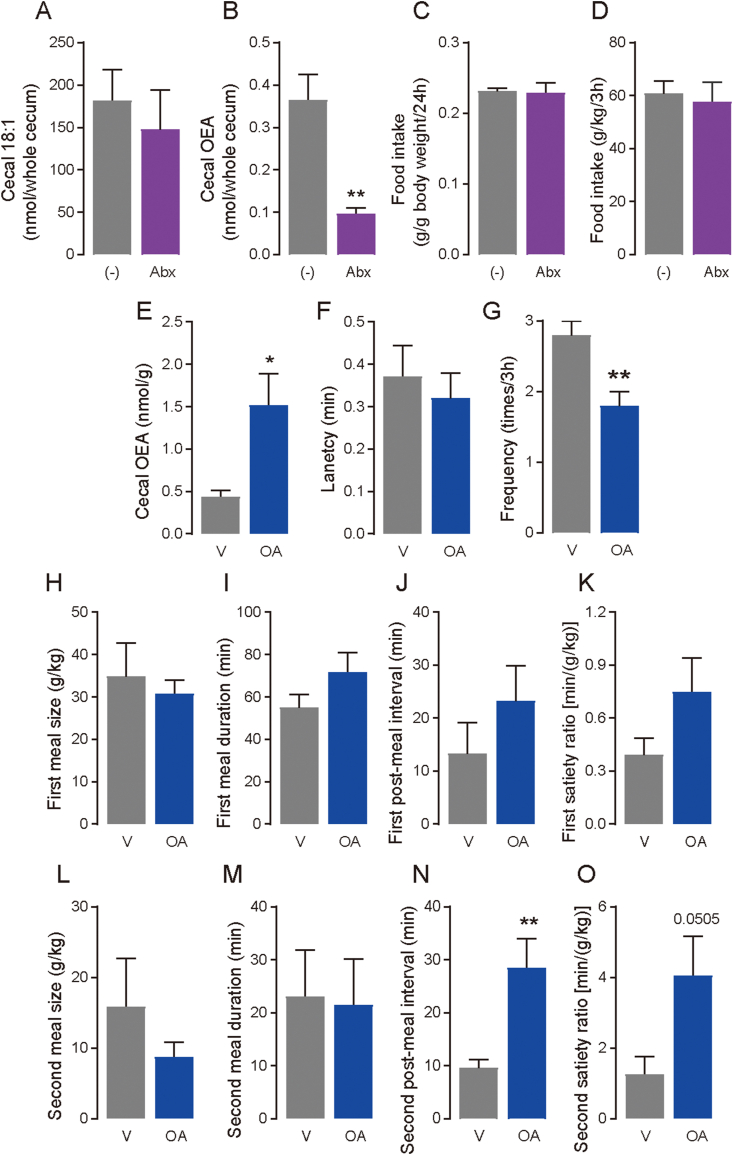
**OEA generated by microbiota possibly modifies feeding behavior**. (A–D) WT mice were given drinking water with/without an antibiotic cocktail for a week. On day 7, cecal lipid levels and food intake were analyzed. (−), control group; Abx, antibiotics group. (A) Cecal 18:1 free fatty acid levels (n = 4), (B) cecal OEA levels (n = 4), (C) food intake for 24 h in FF conditions (n = 4), and (D) food intake for 3 h from the onset of the refeeding after 24 h food deprivation measured by the feeding system (n = 8 for control group, n = 6 for antibiotics group). (E–O) Mice with an implanted large-intestinal catheter were deprived of food for 24 h before infusions. Infusions of oleic acid (OA; 1.0 mg/mice) or vehicle (V; PBS containing 0.3% xanthan gum) were conducted in 30 s and then the mice were placed into the feeding measurement system. In addition, cecal contents were collected 2 h after OA infusion to analyze OEA levels. (E) OEA levels in the cecal contents (n = 4 for vehicle group, n = 3 for OA group). Meal parameters were calculated using raw data, including (F) latency, (G) meal frequency, (H) first meal size, (I) first meal duration, (J) first post-meal interval, (K) first satiety ratio, (L) second meal size, (M) second meal duration, (N) second post-meal interval, and (O) second satiety ratio (n = 5). Data are expressed as means ± SEM. Statistical significance is indicated by asterisks (∗*p* < 0.05, and ∗∗*p* < 0.01). WT, wild type; KO, knockout.

Our previous study showed that microorganisms could convert unabsorbed fatty acids reaching in the distal intestine to unique fatty acid metabolites, such as conjugated fatty acids and hydroxy fatty acids, which have physiological functions [3]. We hypothesized that luminal OEA levels in the large intestine were increased (Figure 5E) because of the synthesis of OA by microorganisms, although it was possible that dietary OEA was accumulated in the luminal contents of the large intestine. To verify this hypothesis, we first quantified OEA levels in the cecal contents of mice administered antibiotics for one week. Cecum weight dramatically increased in antibiotic-treated mice compared with those in mice which were not administered antibiotic (*p* < 0.001; Supplementary Fig. 5A); however, there were no differences in the water level in the cecal content between the groups (Supplementary Fig. 5B). Further, the total number of bacteria was reduced by 99.9% in the cecum of antibiotic-treated mice (*p* < 0.001; Supplementary Fig. 5C). Thus, we analyzed the amounts of OEA and OA (as 18:1 FFA) in the cecum of mice administered antibiotics. Cecal OEA levels were significantly decreased after antibiotic treatment (*p* = 0.0046; Figure 7B), although there was no significant difference in the 18:1 levels between the groups (Figure 7A). These results suggest that the gut microbiota synthesizes OEA from OA that reaches the large intestine. However, antibiotic treatment did not affect food intake during either 24 h under FF conditions (Figure 7C), or RF for 3 h after 24 h of food deprivation (Figure 7D). Antibiotic treatment can lead to many changes in the host, gut microbiota, and their metabolites, including OEA production; therefore, this model might not exhibit OEA-specific effects on food intake in mice. Although food intake did not change, antibiotic treatment affected meal pattern in mice: increased meal frequency (*p* = 0.005; Supplementary Fig. 5E), and reduced average meal size (*p* = 0.0414; Supplementary Fig. 5G), and average meal duration (*p* = 0.0096; Supplementary Fig. 5H), but not latency (Supplemental Fig. 5D), total meal duration (Supplementary Fig. 5F), average meal interval (Supplementary Fig. 5I) and average satiety ratio (Supplementary Fig. 5J), suggesting that antibiotic treatment disrupted the mechanisms underlying satiation.

Next, we examined whether the gut microbiota in the cecum biosynthesizes OEA using OA as a substrate. OA was directly administered into the large intestine of mice through an implanted catheter at the end of ileum after 24 h of food deprivation, and OEA levels were determined in the cecal contents collected 2 h after OA infusion. As a result, cecal OEA levels increased after OA infusion (*p* = 0.0204; Figure 7E), indicating that the microbiota in the large intestine biosynthesizes OEA from OA. Therefore, we next investigated whether OEA biosynthesis from OA induces a similar change in meal patterns as that observed after OEA infusion. The OA infusion did not affect latency (Figure 7F), first meal size (Figure 7H), first meal duration (Figure 7I), first post-meal interval (Figure 7J), or first satiety ratio (Figure 7K). We then analyzed the meal pattern for the second meal, which occurred approximately 90–120 min after OA infusion. OA infusion significantly increased the second post-meal interval (*p* = 0.010; Figure 7N). In contrast, no change was observed in the second meal size (Figure 7L) and second meal duration (Figure 7M), resulting increase of second satiety ratio as a trend (*p* = 0.0505; Figure 7O) and reduction of meal frequency (*p* = 0.0077; Figure 7G). Collectively, these results indicate that microbiota generated OEA using OA as substrate and the OEA induces meal satiety.

## 4 Discussion

The current study revealed that *Gpr119* KO mice exhibited reduced meal satiety for the second and subsequent meals during RF after food deprivation, resulting in increased food intake. In addition, the induction of meal satiety was associated with microbiota-produced OEA derived from OA in the luminal contents of the distal intestine, where *Gpr119* is highly expressed. These findings indicate that intestinal GPR119 modulates host feeding behavior via metabolites, such as OEA, produced by the gut microbiota.

Previous investigations have established that OEA is a satiety factor induced by dietary fat in the proximal intestine [12,24]. Systemic treatment with OEA reduces food intake in rats and mice housed under either FF or RF conditions [19,25,26]. Under FF conditions, OEA regulates food intake by activating PPARα, but not GPR119 or TRPV1, which has been reported as another receptor for OEA [14,27]. In this study, we showed that lack of GPR119 did not affect satiety induced by systematically injecting OEA in mice maintained under either RF or FF conditions. In contrast, infusion of OEA into the large intestine, where *Gpr119* is highly expressed, increased first meal satiety in mice maintained under RF conditions, resulting in decreased food intake. Interestingly, *Gpr119* KO mice exhibited reduced meal satiety for the second and subsequent meals, which occurred 1–2 h after the beginning of feeding. In addition, RF increased OEA levels, which reached EC_50_ [5], in the contents of the distal intestine after 1 and 2 h. Higuchi et al. [10] suggested that disruption of dietary fat absorption in the proximal intestine reduced food intake in 10.13039/100010269WT mice, but not in *Gpr119* KO mice, which support our findings, although they focused on 2-10.13039/100013061OG as a GPR119 ligand. Collectively, intestinal GPR119 senses OEA or other ligands, such as *N*-acyl amides, in the intestinal contents or tissues after meals [18], which induces signals that regulate physiological responses such as feeding behavior.

Antibiotic-induced disruption of the gastrointestinal microbiota revealed that OEA accumulation in the intestinal contents was attributed to the microbiota rather than the diet. This was further confirmed by the results observed after OA infusion into the large intestine. Interestingly, although large-intestinal OEA infusion affected the first meal parameters, further experiments revealed that OEA locally synthesized from OA in the distal intestine induced satiety for the second meal. We expected that OA, which is one of ligands of GPR40 or GRP120, alone would induce satiety; however, under our experimental conditions, large-intestinal infusion of OA did not affect feeding behavior through those receptors because the first meal parameters were not affected. Thus, we predicted that the microbiota-produced OEA is an activator of GPR119 in the large intestine, and the activation results in induction of satiety. Although, antibiotic treatment did not affect food intake in mice, it affected average meal size and meal duration, suggesting that antibiotic treatment alters the mechanisms underlying satiation rather than satiety.

Previous reports suggested that OEA synthesized in the proximal intestine suppresses food intake by recruiting sensory afferents of the vagus nerve, although the detailed mechanism underlying this effect remains unknown [11,13]. GPR119 expressed on the peripheral nerve might not play a role in satiety induced by OEA because intraperitoneal injection of OEA reduced food intake in *Gpr119* KO mice [8]. In addition to the peripheral nerve system, the intestine regulates satiety or satiation by releasing gut hormones, such as GLP-1 and PYY, in response to signals from receptors, including GPR119. Notably, GLP-1 can be released following the activation of GPR119 in L cells by OEA treatment *in vitro* and *in vivo*, which led us to hypothesize that the OEA–GPR119 signal in the large intestine reduces food intake through GLP-1 [15,28]. However, large-intestinal infusion of OEA reduces food intake and increases meal satiety; nevertheless, GLP-1 has been recognized as an endogenous satiation signal [29,30]. Furthermore, under our experimental conditions, plasma GLP-1 levels were not significantly reduced by the deletion of *Gpr119* under FF conditions, suggesting that GPR119 is not the only regulator of GLP-1. However, the deletion of *Gpr119* induced a reduction in the expression of *Gcg*. In addition, Panaro et al. suggested that GLP-1 secretion in acute response to nutrients occurs in the proximal intestine but not in the distal intestine [31]. Therefore, at least under our experimental conditions, GLP-1 secretion is not essential for large intestinal GPR119-induced satiety. In the future, we need to elucidate the detailed mechanisms underlying the GPR119-mediated effects of the microbiota-produced OEA on feeding behavior.

Another interesting finding was that the lack of GPR119 impaired the physiological protective responses against food deprivation. After food deprivation, *Gpr119* KO mice exhibited low blood glucose levels and reduced body weight compared with that in WT mice, suggesting failed hormonal or neuronal responses to food deprivation. *Gpr119* KO mice exhibited low *Gcg* expression in the gut, assuming low levels of glucagon, which can increase the concentration of blood glucose by acting on the liver for glycogenolysis and secretion by alpha-islet cells or the intestine. Increased energy expenditure has been observed in Synaptotagmin-7 KO mice, exhibiting significantly reduced glucagon levels but normal insulin levels [32]. Moreover, several GPR119 agonists have been reported to enhance glucagon secretion during hypoglycemia in *ex vivo* and *in vivo* experiments [33]. Therefore, GPR119 might be necessary for glucagon secretion as well as insulin secretion, resulting in reduced glucose levels and increased food intake in *Gpr119* KO mice. Interestingly, food deprivation increased *Gpr119* expression in the cecum of mice. These results require further investigation to determine the glucagon-mediated physiological function of GPR119.

Subchronic OEA treatment lowed body weight and hyperlipidemia in obese rodents as resulting of feeding modification [14]. In addition, OEA administration caused a significant fat mass reduction and enhanced energy expenditure in rats [34]. However, lack of *Gpr119* resulted in lower bodyweight in seven-week-old mice, with no changes in food intake. Using another *Gpr119* KO mice line, the absence of *Gpr119* led to lower body weight and fat mass compared to WT at 40 weeks of age [8]. Therefore, we measured energy expenditure in mice. Our findings showed that *Gpr119* KO had high adjusted energy expenditure compared with WT mice. In *Gpr119* KO mice, energy expenditure was higher in the dark cycle, especially after the main meal (20:00–23:00), while no changes in the respiratory quotient and locomotor activity were observed. These results suggest the enhancement of diet-induced thermogenesis in *Gpr119* KO mice for which a detailed investigation could be interesting to reveal a potential new function of GPR119.

It is not yet clear if there are sex differences regarding GPR119 function in either animal or clinical studies. One study revealed that *Gpr119* expression was higher in the anterior eye of female mice than that in the anterior eye of male mice [35]. Additionally, GPR119 activation by 2-OG reduced intraocular pressure only female mice [35]. In the intestine, GPR119 function may be similar in males and females because there were no sex differences in plasma GLP-1 levels in mice [28]. Although it is not a functional comparison, GPR119 was expressed in testes, but not ovaries, in human samples [36]. It would be worthwhile to investigate potential sex-dependent functions of GPR119 in the future.

In conclusion, our study shows that GPR119 in the large intestine induces satiety by sensing OEA produced by the gut microbiota. GPR119 has already been pharmacologically targeted for obesity and diabetes because it stimulates insulin secretion by directly acting on pancreatic beta cells or through incretin secretion by gut enteroendocrine cells. Therefore, strategies aimed at enhancing the activation of GPR119, such as modulation of microbiota composition to increase OEA production or inhibition of OEA degradation, may help to induce meal satiety in obesity and other eating disorders.

## Author contributions

Conceptualization, MI, IK; Methodology and Data curation**,** MI, TH, HT, KW, and AN; Formal analysis, MI, TH, and KI; Writing- Original draft and Visualization, MI; Reviewing & editing, MI, TH, HT, KW, AN and IK. Supervision, IK. All authors have finalized and approved the manuscript.

## Figures and Tables

**Figure:**
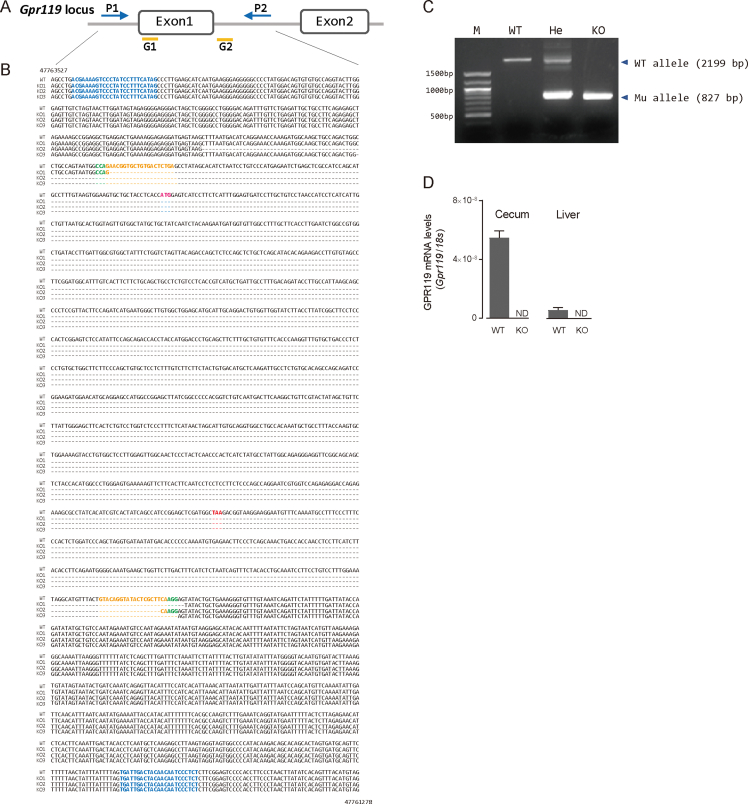
**Supplementary Figure 1. CRISPR/Cas9-mediated *Gpr119* deletion in mice. (A) Schematic diagram of the gRNA locations (G1 and G2) at the *Gpr119* locus. Arrows P1 and P2 indicate the primer locations for genotyping. (B) Sequence alignment of *Gpr119* in WT and *Gpr119* KO mice (KO1–3). KO1 was used as *Gpr119* KO mice line in this study. Genotyping primers highlighted in blue. Genomic protospacer adjacent motif (PAM) sequences are highlighted in green, and guide RNA sequences are in orange. Start and stop codons are highlighted in pink and red, respectively. (C) PCR-based genotyping of *Gpr119* KO mice. The WT locus yields an amplicon of 2199 bp, whereas mutant locus yields an amplicon of 827 bp (KO1). (D) Confirmation of lack of *Gpr119* expression in the cecum and liver of *Gpr119* KO mice. ND, not detected. Data are expressed as means ± SEM (n = 4). WT, wild type; KO, knockout**.

**Figure:**
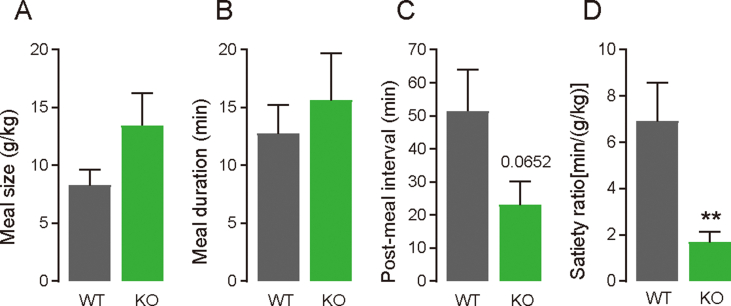
**Supplementary Figure 2. Meal patterns of the second meal of WT and *Gpr119* KO mice**. After 24 h food-deprivation, WT or *Gpr119* KO mice were individually placed into a cage with food in the feeding behavior analysis system, and test sessions were recorded for up to 5 h. Meal parameters were analyzed in the first 3 h. Parameters of second meal were showed in this figure: (A) second meal size, (B) second meal duration, (C) second post-meal interval, and (D) satiety ratio of second meal (n = 6 for WT and n = 7 for KO). Data are expressed as means ± SEM. Statistical significance is indicated by asterisks (∗∗*p* < 0.01). WT, wild type; KO, knockout.

**Figure:**
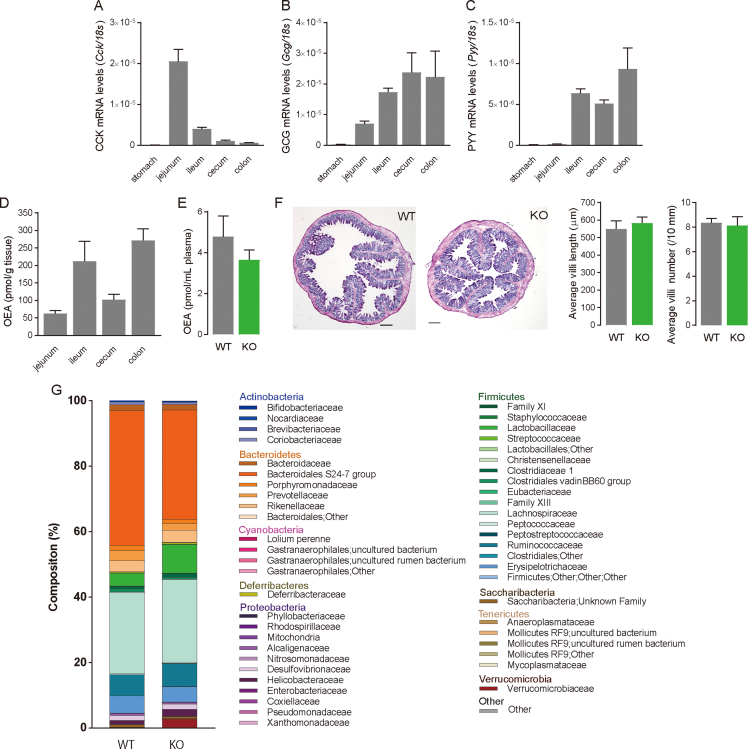
**Supplementary Figure 3. Assessment of intestinal mRNA expression of gut hormones, OEA levels of intestinal tissues and plasma, cecal histology, and fecal microbial composition in WT and *Gpr119* KO mice.** Expression levels of (A) *Cck* (B) *Gcg* and (C) *Pyy* in the intestinal segments of seven-week-old WT mice (n = 3 for stomach, cecum, and colon, n = 4 for jejunum and ileum). (D) OEA levels in the intestinal segments of seven-week-old WT mice (n = 4). (E) Plasma OEA levels in seven-week-old WT and *Gpr119* KO mice (n=5 for WT, and n = 4 for KO). (F) Representative hematoxylin and eosin staining of cecal tissue of seven-week-old WT and *Gpr119* KO mice. Villi length (μm) and number of villi (number/10 mm serosa length) were assessed in staining images. Scale bar = 200 μm. (G) Relative composition of fecal microbiota at the family level in seven-week-old WT and *Gpr119* KO mice (Average of five samples). Data are expressed as means ± SEM. WT, wild type; KO, knockout.

**Figure:**
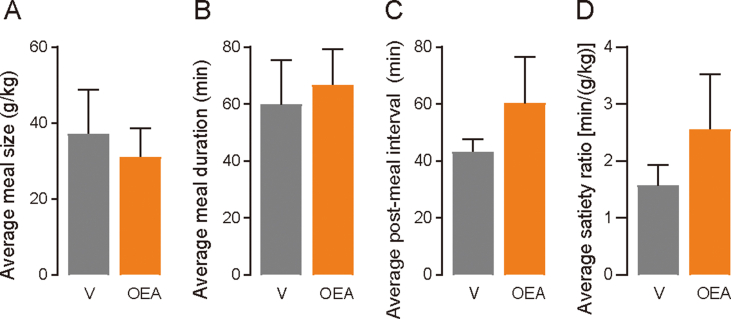
**Supplementary Figure 4.Average meal parameters in mice infused with OEA.** Mice implanted with a catheter at the end of the ileum were deprived of food for 24 h before infusions. Infusions of OEA (0.4 mg/mice, OEA in PBS containing 0.3% xanthan gum and 10% DMSO) or vehicle (V; PBS containing 0.3% xanthan gum and 10% DMSO) were conducted in 30 s. Mice were placed into the feeding measurement system, and test sessions were recorded. Meal parameters were calculated using raw data of the first two hours, including (A) average meal size, (B) average meal duration, (C) average post-meal interval, and (D) average satiety ratio. Other parameters calculated are showed in Figure 6. Data are expressed as means ± SEM (n=5).

**Figure:**
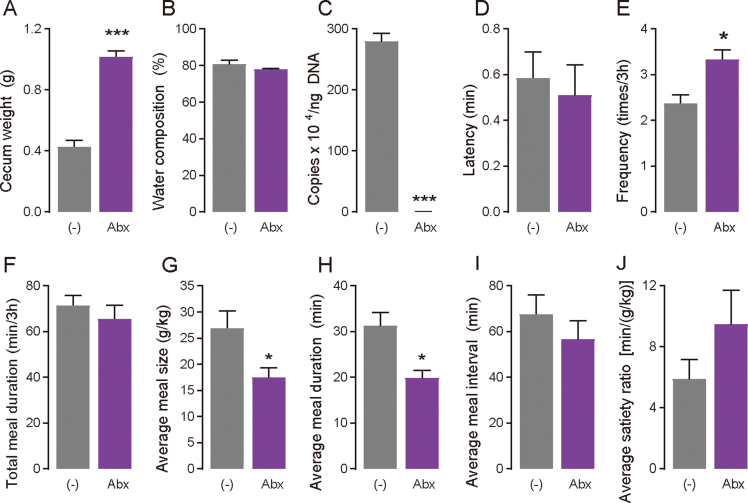
**Supplementary Figure 5.Meal pattern changes in the mice treated with antibiotics.** WT mice were given drinking water for 7 days with/without an antibiotic cocktail. (A) Weight and (B) water composition of the cecum of mice after 7 days of treatment (n=4). (C) Total bacteria number was measured in the cecum of mice after 7 days of antibiotic treatment (n=5). After 7 days of antibiotics treatment, the mice were deprived of food for 24 h and then placed into the feeding measurement system. Meal parameters were calculated using raw data of the first 3 h, including (D) latency (n=5), (E) meal frequency, (F) total meal duration, (G) average meal size, (H) average meal duration, (I) average meal interval, and (J) average satiety ratio (E-J: n=8 for control group, n=6 for antibiotics group). (-), control group; Abx, antibiotics group. Data are expressed as means ± SEM. Food intake of this data set is showed in Figure 7D. Statistical significance is indicated by asterisks (∗*p* < 0.05 and ∗∗∗*p* < 0.001).

## Data Availability

Data will be made available on request.

## References

[1] Kimura I., Ichimura A., Ohue-Kitano R., Igarashi M. (2020). Free fatty acid receptors in health and disease. Physiological Reviews.

[2] Canals M., Poole D.P., Veldhuis N.A., Schmidt B.L., Bunnett N.W. (2019). G-Protein-Coupled receptors are dynamic regulators of digestion and targets for digestive diseases. Gastroenterology.

[3] Miyamoto J., Igarashi M., Watanabe K., Karaki S.I., Mukouyama H., Kishino S. (2019). Gut microbiota confers host resistance to obesity by metabolizing dietary polyunsaturated fatty acids. Nature Communications.

[4] Hosomi K., Kiyono H., Kunisawa J. (2020). Fatty acid metabolism in the host and commensal bacteria for the control of intestinal immune responses and diseases. Gut Microbes.

[5] Hansen H.S., Rosenkilde M.M., Holst J.J., Schwartz T.W. (2012). GPR119 as a fat sensor. Trends in Pharmacological Sciences.

[6] Neunlist M., Schemann M. (2014). Nutrient-induced changes in the phenotype and function of the enteric nervous system. Journal of Physiology.

[7] Psichas A., Reimann F., Gribble F.M. (2015). Gut chemosensing mechanisms. Journal of Clinical Investigation.

[8] Lan H., Vassileva G., Corona A., Liu L., Baker H., Golovko A. (2009). GPR119 is required for physiological regulation of glucagon-like peptide-1 secretion but not for metabolic homeostasis. Journal of Endocrinology.

[9] Panaro B.L., Flock G.B., Campbell J.E., Beaudry J.L., Cao X., Drucker D.J. (2017). Beta-Cell inactivation of Gpr119 unmasks incretin dependence of GPR119-mediated glucoregulation. Diabetes.

[10] Higuchi S., Ahmad T.R., Argueta D.A., Perez P.A., Zhao C., Schwartz G.J. (2020). Bile acid composition regulates GPR119-dependent intestinal lipid sensing and food intake regulation in mice. Gut.

[11] Piomelli D. (2013). A fatty gut feeling. Trends in Endocrinology and Metabolism.

[12] Fu J., Astarita G., Gaetani S., Kim J., Cravatt B.F., Mackie K. (2007). Food intake regulates oleoylethanolamide formation and degradation in the proximal small intestine. Journal of Biological Chemistry.

[13] Schwartz G.J., Fu J., Astarita G., Li X., Gaetani S., Campolongo P. (2008). The lipid messenger OEA links dietary fat intake to satiety. Cell Metabolism.

[14] Fu J., Oveisi F., Gaetani S., Lin E., Piomelli D. (2005). Oleoylethanolamide, an endogenous PPAR-alpha agonist, lowers body weight and hyperlipidemia in obese rats. Neuropharmacology.

[15] Lauffer L.M., Iakoubov R., Brubaker P.L. (2009). GPR119 is essential for oleoylethanolamide-induced glucagon-like peptide-1 secretion from the intestinal enteroendocrine L-cell. Diabetes.

[16] Chen Z., Guo L., Zhang Y., Walzem R.L., Pendergast J.S., Printz R.L. (2014). Incorporation of therapeutically modified bacteria into gut microbiota inhibits obesity. Journal of Clinical Investigation.

[17] Dosoky N.S., Guo L., Chen Z., Feigley A.V., Davies S.S. (2018). Dietary fatty acids control the species of N-Acyl-Phosphatidylethanolamines synthesized by therapeutically modified bacteria in the intestinal tract. ACS Infectious Diseases.

[18] Cohen L.J., Esterhazy D., Kim S.H., Lemetre C., Aguilar R.R., Gordon E.A. (2017). Commensal bacteria make GPCR ligands that mimic human signalling molecules. Nature.

[19] Gaetani S., Oveisi F., Piomelli D. (2003). Modulation of meal pattern in the rat by the anorexic lipid mediator oleoylethanolamide. Neuropsychopharmacology.

[20] Igarashi M., Watanabe K., Tsuduki T., Kimura I., Kubota N. (2019). NAPE-PLD controls OEA synthesis and fat absorption by regulating lipoprotein synthesis in an in vitro model of intestinal epithelial cells. The FASEB Journal.

[21] Kimura I., Ozawa K., Inoue D., Imamura T., Kimura K., Maeda T. (2013). The gut microbiota suppresses insulin-mediated fat accumulation via the short-chain fatty acid receptor GPR43. Nature Communications.

[22] Hirasawa A., Tsumaya K., Awaji T., Katsuma S., Adachi T., Yamada M. (2005). Free fatty acids regulate gut incretin glucagon-like peptide-1 secretion through GPR120. Nature Medicine.

[23] Svendsen B., Pedersen J., Albrechtsen N.J., Hartmann B., Torang S., Rehfeld J.F. (2015). An analysis of cosecretion and coexpression of gut hormones from male rat proximal and distal small intestine. Endocrinology.

[24] Bowen K.J., Kris-Etherton P.M., Shearer G.C., West S.G., Reddivari L., Jones P.J.H. (2017). Oleic acid-derived oleoylethanolamide: a nutritional science perspective. Progress in Lipid Research.

[25] Rodriguez de Fonseca F., Navarro M., Gomez R., Escuredo L., Nava F., Fu J. (2001). An anorexic lipid mediator regulated by feeding. Nature.

[26] Oveisi F., Gaetani S., Eng K.T., Piomelli D. (2004). Oleoylethanolamide inhibits food intake in free-feeding rats after oral administration. Pharmacological Research.

[27] Lo Verme J., Gaetani S., Fu J., Oveisi F., Burton K., Piomelli D. (2005). Regulation of food intake by oleoylethanolamide. Cellular and Molecular Life Sciences.

[28] Moss C.E., Glass L.L., Diakogiannaki E., Pais R., Lenaghan C., Smith D.M. (2016). Lipid derivatives activate GPR119 and trigger GLP-1 secretion in primary murine L-cells. Peptides.

[29] Steinert R.E., Beglinger C., Langhans W. (2016). Intestinal GLP-1 and satiation: from man to rodents and back. International Journal of Obesity.

[30] Krieger J.P. (2020). Intestinal glucagon-like peptide-1 effects on food intake: physiological relevance and emerging mechanisms. Peptides.

[31] Panaro B.L., Yusta B., Matthews D., Koehler J.A., Song Y., Sandoval D.A. (2020). Intestine-selective reduction of Gcg expression reveals the importance of the distal gut for GLP-1 secretion. Molecular Metabolism.

[32] Lou P.H., Gustavsson N., Wang Y., Radda G.K., Han W. (2011). Increased lipolysis and energy expenditure in a mouse model with severely impaired glucagon secretion. PLoS One.

[33] Li N.X., Brown S., Kowalski T., Wu M., Yang L., Dai G. (2018). GPR119 agonism increases glucagon secretion during insulin-induced hypoglycemia. Diabetes.

[34] Suárez J., Rivera P., Arrabal S., Crespillo A., Serrano A., Baixeras E. (2014). Oleoylethanolamide enhances β-adrenergic-mediated thermogenesis and white-to-brown adipocyte phenotype in epididymal white adipose tissue in rat. Dis Model Mech.

[35] Miller S., Hu S.S., Leishman E., Morgan D., Wager-Miller J., Mackie K. (2017). A GPR119 signaling system in the murine eye regulates intraocular pressure in a sex-dependent manner. Investigative Ophthalmology & Visual Science.

[36] Odori S., Hosoda K., Tomita T., Fujikura J., Kusakabe T., Kawaguchi Y. (2013). GPR119 expression in normal human tissues and islet cell tumors: evidence for its islet-gastrointestinal distribution, expression in pancreatic beta and alpha cells, and involvement in islet function. Metabolism.

